# Prognostic Model for Predicting Overall and Cancer-Specific Survival Among Patients With Cervical Squamous Cell Carcinoma: A SEER Based Study

**DOI:** 10.3389/fonc.2021.651975

**Published:** 2021-07-14

**Authors:** Zhuolin Li, Yao Lin, Bizhen Cheng, Qiaoxin Zhang, Yingmu Cai

**Affiliations:** ^1^ Department of Clinical Laboratory, The First Affiliated Hospital of Shantou University Medical College, Guangdong, China; ^2^ Department of Plastic Surgery and Burn Center, The Second Affiliated Hospital of Shantou University Medical College, Guangdong, China

**Keywords:** nomogram, cervical cancer, cancer-specific survival, overall survival, prognosis

## Abstract

**Background:**

Cervical squamous cell carcinoma (CSCC) is the most common histological subtype of cervical cancer. The purpose of this study was to assess prognostic factors and establish personalized risk assessment nomograms to predict overall survival (OS) and cancer-specific survival (CSS) in CSCC patients.

**Methods:**

CSCC patients diagnosed between 1988 and 2015 were identified in the Surveillance, Epidemiology, and End Results (SEER) database. Univariate and multivariate Cox proportional hazard regression models were applied to select meaningful independent predictors and construct predictive nomogram models for OS and CSS. The concordance index (C-index), calibration curve, and receiver operating characteristic (ROC) curve were used to determine the predictive accuracy and discriminability of the nomogram.

**Results:**

A total cohort (n=17962) was randomly divided into a training cohort (n=11974) and a validation cohort (n=5988). Age, race, histologic grade, clinical stage, tumor size, chemotherapy and historic stage were assessed as common independent predictors of OS and CSS. The C-index value of the nomograms for predicting OS and CSS was 0.771 (95% confidence interval 0.762-0.780) and 0.786 (95% confidence interval 0.777-0.795), respectively. Calibration curves of the nomograms indicated satisfactory consistency between nomogram prediction and actual survival for both 3-year and 5-year OS and CSS.

**Conclusion:**

We constructed nomograms that could predict 3- and 5-year OS and CSS of CSCC patients. These nomograms showed good performance in prognostic prediction and can be used as an effective tool to evaluate the prognosis of CSCC patients, thus contributing to clinical decision making and individualized treatment planning.

## Introduction

Cervical cancer is the fourth most common cancer in women worldwide and is especially common in low- and middle-income countries (1). The most common cause for the occurrence of cervical cancer is a persistent infection with high-risk subtypes of the human papilloma virus (HPV) (2). Despite the fact that cervical cancer incidence and mortality rates have been in decline in high-income countries over the past 30 years as a result of the implementation of HPV vaccination and screening programs, cervical cancer continues to be a major public health challenge (3). In addition, cervical cancer remains the second leading cause of cancer death among women 20 to 39 years of age, causing 9 deaths per week in this age group (4). It is now generally accepted that clinical stage is a reliable prognostic indicator for patients with cervical carcinoma (5). There are currently two main clinical staging schemes: the American Joint Committee on Cancer’s Cancer Staging Manual 7th edition (AJCC 7th) and the 2018 International Federation of Gynecology and Obstetrics Staging Guidelines (FIGO 2018). In the United States, the overall 5-year survival rates of stages I, II and III cervical cancer are about 84.1% to 87.0%, 51.4% to 60.5% and 33.9% to 44.7%, respectively (6). Histological type is also an important indicator of prognosis. Histologically, cervical cancer is mainly classified into two histological types, adenocarcinoma and squamous cell carcinoma, among which squamous cell carcinoma is more common, accounting for about 90% of all cases (1). In this article, we focused on cervical squamous cell carcinoma (CSCC). Clinical staging is mainly based on the tumor size in the cervix or its extension into the pelvis, without taking into account many other important prognostic factors, such as age, race, or treatment model. Thus, it is obvious that clinical stage is still insufficient to predict the prognosis of a CSCC patient. Therefore, it is necessary to establish a more complete prognostic evaluation scheme.

Although CSCC causes a far greater health concern in less developed than in more developed countries, the information discussed here is based more on research findings in more developed countries. Our data is based on the Surveillance, Epidemiology, and End Results (SEER) database, a National Cancer Institute-funded program collecting data on cancer diagnoses, treatment and survival, and covers approximately 30% of the US population (7). It is an important population-based resource that collects demographic, clinical, and outcome information for all cancers, and is freely available to researchers (7). On this basis, we use a statistical prediction model to build a nomogram, which is a simple graphical representation that can generate a numerical probability of a clinical event for an individual patient (8). As a common tool for prognostic assessment in oncology and medicine, nomogram is able to generate individualized predictions by integrating multiple prognostic and determinant variables, enabling its use in the identification and stratification of patients for personalized medicine (8, 9). Our study derived and validated a prognostic nomogram to predict overall survival (OS) and cancer-specific survival (CSS), for CSCC patients who registered between 1988 and 2015 in the SEER database, to aid in clinical decision making and assist in ongoing work. Compared with other studies using nomograms to study the prognosis of cervical cancer survival (10–14), our sample size is larger and spans a longer time line, which improves the universality of the scheme. More importantly, we predict not only OS, but also CSS, and assessed the performance of our statistical prediction model internally and externally from three aspects, the C-index (Harrell’s concordance index), calibration curve and ROC (receiver operating characteristic) curve, making our conclusions more certain.

## Materials and Methods

### Data Source and Study Population

Information of patients who had been diagnosed with cervical squamous cell carcinoma between 1988 and 2015 was obtained from the Surveillance, Epidemiology, and End Results (SEER) database by SEER*Stat software (version 8.3.6.1; https://seer.cancer.gov/seerstat/).The SEER database data was derived from 18 cancer registry databases (with additional treatment fields) (15). We obtained signed authorization and permission from the SEER program to access and use the data, and followed the agreement throughout the process to protect patient privacy.

The following were the inclusion criteria: (1) site recode ICD-O-3/WHO2008: Cervix Uteri, (2) histologic type ICD-O-3: 8070-8078, (3) year of diagnosis: 1988-2015, (4) known cause of death and survival time.

The following were the exclusion criteria: (1) unknown diagnostic method, (2) unknown histologic grade, (3) unknown cause of death, (4) unknown AJCC stage, (5) unknown race, (6) unknown metastasis, (7) unknown tumor size, (8) no first tumor.

### Data Collection

**Figure 1:**
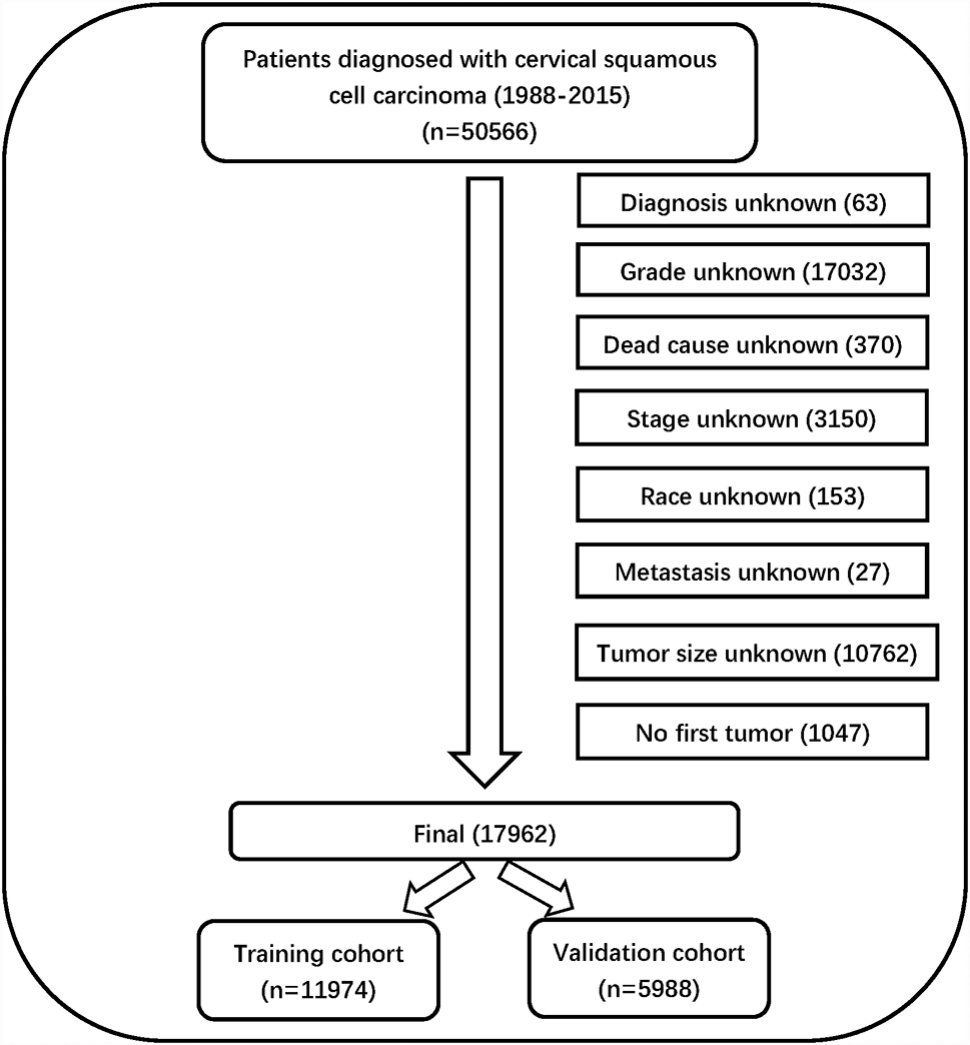
Chart of the data filtering process and grouping information.

Information on 50,566 patients was collected from the SEER database. The data processing flowchart is shown in **Figure 1**. Overall, 17962 patients with CSCC were enrolled in our study, all of them were randomly divided into a training cohort (n = 11974) and validation cohort (n = 5988) at a ratio of 2:1.

Variables for each patient included patient ID, age, race, year of diagnosis, histology grade, clinical stage, tumor size, metastasis status, pathological subtype, historic stage, radiation recode, chemotherapy recode, diagnosis confirmation recode, cause-specific death classification, vital status recode, and survival time. The primary endpoint of this study included OS and CSS.

OS was defined as the time from the date of diagnosis to death due to any cause (for patients who had been lost to follow-up prior to death, the last follow-up time was usually calculated as the time of death). CSS was defined as the time from the date of diagnosis to the date of death caused by cervical squamous cell carcinoma.

### Statistical Analysis

**Figure 2:**
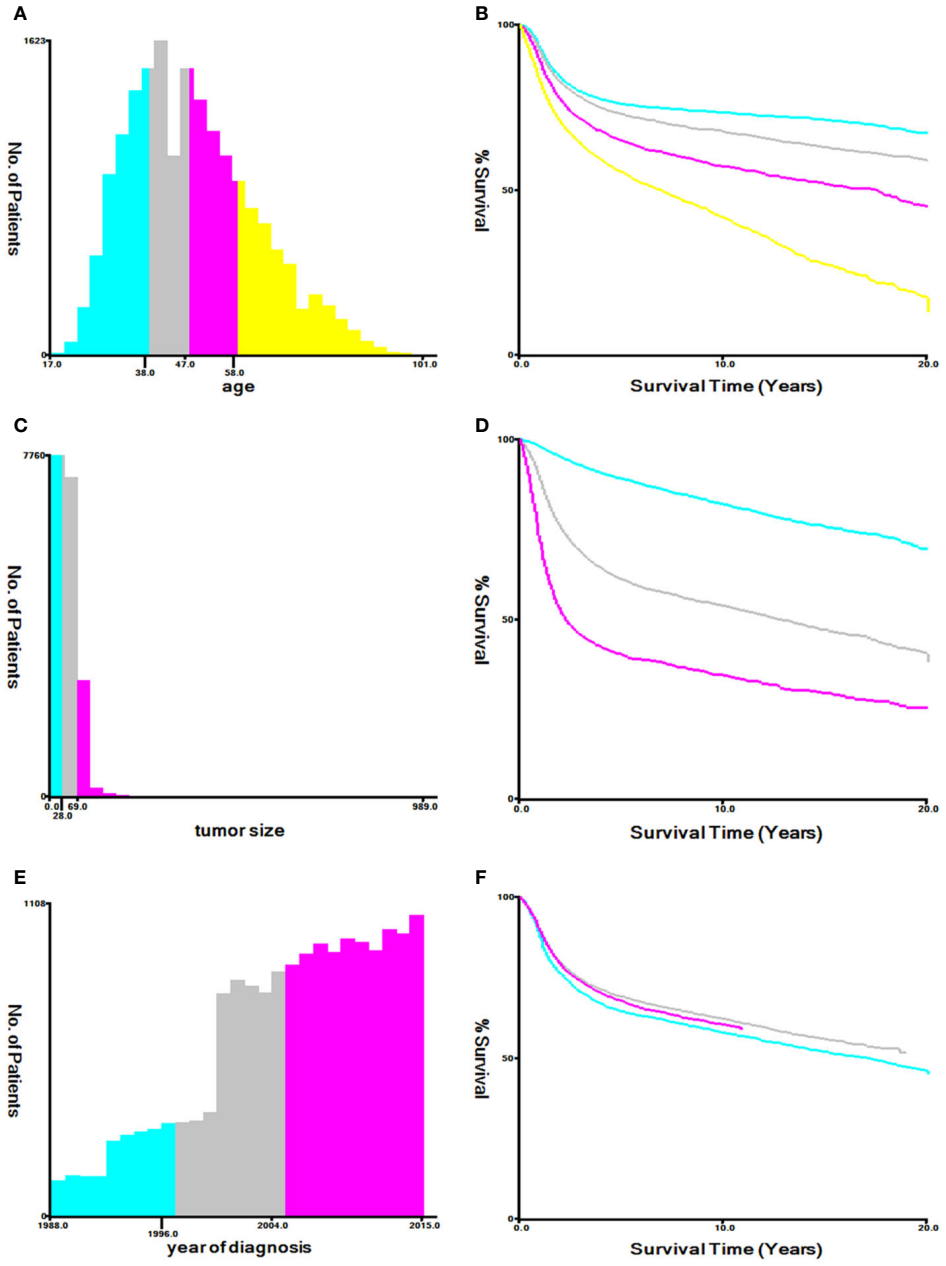
Optimal cutoff values ​​for patient’s age **(A, B)**, tumor size **(C, D)**, and year of diagnosis **(E, F)** through X-tile software analysis. The optimal age cut-off values ​​determined by overall survival were 38, 47, and 58 years. The optimal tumor size cutoff values ​​determined by overall survival were 28 mm and 69 mm. The optimal year of diagnosis cutoff values ​​determined by overall survival were 1996 and 2004.

Using the Kaplan–Meier method in X-tile software to evaluate the optimal cut-off values ​​for patient age, tumor size and year of diagnosis (16). The optimal cut-off values for age were 38-, 47-, and 58-years; The optimal cut-off values for tumor size were 28, and 69 mm; The optimal cut-off values for year of diagnosis were 1996, and 2004 (**Figure 2**). Variables that were statistically significant in the univariate Cox regression model were analyzed in the multivariate Cox regression model, and multivariate proportional hazard models were used to identify independent prognostic factors associated with OS and CSS and the hazard ratio and 95% confidence interval. Analysis items with P < 0.05 were considered statistically significant. The chi-square test and Cox regression analysis were performed using SPSS statistical software package version 23.0 (Chicago, IL, USA). The prognostic nomograms were constructed from the results of the multivariate Cox regression analysis using the training cohort, and it was used to predict the 3- and 5-year OS and CSS by representing the sum of points for each variable (8). The concordance index (C-index) was used to evaluate the exact prognostic values of the prognostic model (17). The receiver operating characteristic (ROC) curve was used to evaluate the precision of the nomograms for the 3-year and 5-year OS and CSS (18). The calibration curves in this study showed the predicted probability between the actual and predicted nomograms of 3- and 5-year OS and CSS (19). Nomograms, ROC curves and the calibration curves were constructed and adjusted using R version 4.0.2 software in RStudio.

## Results

### Patient Baseline Characteristics

Eventually, after using the inclusion and exclusion criteria, a total of 17962 out of 50566 patients with CSCC between 1988 and 2015 were enrolled from the SEER database. All of them were randomly divided into a training cohort (n = 11974) and validation cohort (n = 5988) at a ratio of 2:1. The patients’ baseline characteristics between the training and validation cohorts in our study are summarized in **Table 1**.

**Table 1:** Baseline characteristics of patients with cervical squamous cell carcinoma in the training cohort and validation cohort.

Variables	Training Cohort		Validation Cohort		Total		P
Age, n, %							0.482
≤38	3184	26.6	1585	26.5	4769	26.6	
39-47	3137	26.2	1511	25.2	4648	25.9	
48-58	2823	23.6	1440	24.0	4263	23.7	
≥59	2830	23.6	1452	24.2	4282	23.83	
Race, n, %							0.496
White	9000	75.2	4460	74.5	13460	74.9	
Black	1692	14.1	854	14.3	2546	14.2	
Other	1282	10.7	674	11.3	1956	10.9	
Year of diagnosis, n, %							0.77
≤1996	1353	11.3	677	11.3	2030	11.3	
1997-2004	3442	28.7	1691	28.2	5133	28.6	
≥2005	7179	60.0	3620	60.5	10799	60.1	
Histologic grade, n, %							0.448
Grade I	912	7.6	420	7.0	1332	7.4	
Grade II	5383	45.0	2692	45.0	8075	45.0	
Grade III	5460	45.6	2773	46.3	8233	45.8	
Grade IV	219	1.8	103	1.7	322	1.8	
Clinical stage, n, %							0.646
Stage I	5533	46.2	2714	45.3	8247	45.9	
Stage II	1916	16.0	968	16.2	2884	16.1	
Stage III	3201	26.7	1615	27.0	4816	26.8	
Stage IV	1324	11.1	691	11.5	2015	11.2	
Tumor size, n, %							0.306
≤28	4327	36.1	2129	35.6	6456	35.9	
29-69	5691	47.5	2916	48.7	8607	47.9	
≥70	1956	16.3	943	15.7	2899	16.1	
Metastasis, n, %							0.562
M0	10878	90.8	5424	90.6	16302	90.8	
M1	1096	9.2	564	9.4	1660	9.2	
Radiotherapy, n, %							0.88
No/Unknown	4361	36.4	2174	36.3	6535	36.4	
Yes	7613	63.6	3814	63.7	11427	63.6	
Chemotherapy, n, %							0.358
No/Unknown	6178	51.6	3046	50.9	9224	51.4	
Yes	5796	48.4	2942	49.1	8738	48.6	
Historic stage, n, %							0.408
Localized	5332	44.5	2609	43.6	7941	44.2	
Regional	5484	45.8	2775	46.3	8259	46.0	
Distant	1158	9.7	604	10.1	1762	9.8	

The number of patients ≤38-, 39-47-, 48-58-, ≥59-years of age were 4,769 (26.6%), 4,648 (25.9%), 4,263 (23.7%), and 4,282 (23.8%). There were 13,460 (74.9%) Caucasians, 2,546 (14.2%) of African descent, and 1,956 (10.9%) other races; 2,030 (11.3%) patients were diagnosed before 1997, 5,133 (28.6%) between 1997 and 2004, and 10,799 (60.1%) after 2005. The numbers of people with histological grades G1, G2, G3, and G4 were 1,332 (7.4%), 8,075 (45.0%), 8,233 (45.8%), and 322 (1.8%). The numbers of people in clinical stages I, II, III, and IV were 8,247 (45.9%), 2,884 (16.1%), 4,816 (26.8%), and 2,015 (11.2%), respectively. Those with tumor diameter ≤28, 29–69, and ≥70 mm were 6,456 (35.9%), 8,607 (47.9%), and 2,899 (16.2%), and the metastatic status of M0 and M1 were 16,302 (90.8%) and 1,660 (9.2%), respectively; The number of patients with and without radiotherapy were, respectively, 11,427 (63.6%) and 6,535 (36.4%); There were 8,738 (48.6%) and 9,224 (51.4%) patients in the chemotherapy group and non-chemotherapy group, respectively; and those historic stages with localized, regional, and distant tumors were, respectively, 7,941 (44.2%), 8,259 (46.0%), and 1,762 (9.8%). The chi-square test results for these variables between the training and validation cohorts were all P > 0.05.

### Cox Regression Analyses of Variables for OS and CSS

In the univariate Cox regression, except for “other” in the race category, other variables were all significant in OS and CSS, respectively (P < 0.05) (**Table 2**). Based on the variables identified by univariate analysis, multivariate Cox regression analyses of OS and CSS were constructed. In the multivariate Cox regression for OS, the independent prognostic factors included age, race, histology grade, clinical stage, tumor size, historic stage, radiotherapy and chemotherapy. In the multivariate Cox regression for CSS, the independent prognostic factors included age, race, histology grade, clinical stage, tumor size, historic stage and chemotherapy (**Table 3**).

**Table 2:** Univariate Cox regression analysis of overall survival and cancer-specific survival in cervical squamous cell carcinoma (training cohort).

Variables	Overall survival			Cancer-specific survival		
	HR	95%CI	P	HR	95%CI	P
**Age**						
≤38	**Reference**			**Reference**		
39-47	1.265	1.148-1.394	<0.001*	1.178	1.062-1.306	0.002
48-58	1.796	1.634-1.973	<0.001*	1.561	1.410-1.727	<0.001*
≥59	2.804	2.566-3.064	<0.001*	1.837	1.662-2.030	<0.001*
**Race**						
White	**Reference**			**Reference**		
Black	1.381	1.274-1.498	<0.001*	1.372	1.251-1.504	<0.001*
Other	0.957	0.864-1.061	0.403	0.943	0.838-1.061	0.327
**Year of diagnosis**						
≤1996	**Reference**			**Reference**		
1997-2004	0.881	0.803-0.966	0.007	0.853	0.766-0.950	0.004
≥2005	0.895	0.817-0.981	0.017	0.862	0.778-0.955	0.004
**Histologic grade**						
Grade I	**Reference**			**Reference**		
Grade II	1.580	1.357-1.840	<0.001*	1.853	1.535-2.236	<0.001*
Grade III	2.141	1.842-2.489	<0.001*	2.609	2.167-3.142	<0.001*
Grade IV	2.078	1.607-2.687	<0.001*	2.588	1.924-3.481	<0.001*
**Clinical stage**						
Stage I	**Reference**			**Reference**		
Stage II	2.688	2.445-2.956	<0.001*	3.188	2.833-3.588	<0.001*
Stage III	3.531	3.255-3.832	<0.001*	4.869	4.404-5.384	<0.001*
Stage IV	10.373	9.474-11.356	<0.001*	15.028	13.503-16.726	<0.001*
**Tumor size**						
≤28	**Reference**			**Reference**		
29-69	3.347	3.071-3.648	<0.001*	4.724	4.219-5.290	<0.001*
≥70	6.276	5.699-6.912	<0.001*	9.692	8.586-10.942	<0.001*
**Metastasis**						
M0	**Reference**			**Reference**		
M1	5.252	4.857-5.678	<0.001*	6.193	5.700-6.729	<0.001*
**Radiotherapy**						
No/Unknown	**Reference**			**Reference**		
Yes	2.675	2.482-2.884	<0.001*	3.037	2.775-3.323	<0.001*
**Chemotherapy**						
No/Unknown	**Reference**			**Reference**		
Yes	1.842	1.731-1.961	<0.001*	2.161	2.011-2.323	<0.001*
**Historic stage**						
Localized	**Reference**			**Reference**		
Regional	3.367	3.118-3.636	<0.001*	4.481	4.064-4.941	<0.001*
Distant	10.999	10.001-12.096	<0.001*	16.240	14.513-18.173	<0.001*

HR, hazard Ratio; CI, confidence interval; *P < 0.05 were considered statistically significant.

**Table 3:** Multivariate Cox regression analysis of overall survival and cancer-specific survival in cervical squamous cell carcinoma (training cohort).

Variables	Overall survival			Cancer-specific survival		
	HR	95%CI	P	HR	95%CI	P
**Age**						
≤38	**Reference**			**Reference**		
39-47	1.073	0.974-1.183	0.153	0.974	0.878-1.081	0.623
48-58	1.317	1.197-1.449	<0.001*	1.076	0.971-1.193	0.161
≥59	2.093	1.911-2.293	<0.001*	1.296	1.170-1.436	<0.001*
**Race**						
White	**Reference**			**Reference**		
Black	1.319	1.216-1.431	<0.001*	1.300	1.185-1.426	<0.001*
Other	0.831	0.750-0.922	<0.001*	0.861	0.764-0.969	0.013
**Year of diagnosis**						
≤1996	**Reference**			**Reference**		
1997-2004	0.993	0.902-1.093	0.888	0.943	0.843-1.054	0.303
≥2005	0.961	0.872-1.060	0.429	0.913	0.817-1.019	0.105
**Histologic grade**						
Grade I	**Reference**			**Reference**		
Grade II	1.137	0.975-1.325	0.102	1.224	1.013-1.478	0.036
Grade III	1.275	1.095-1.484	0.002	1.391	1.154-1.678	<0.001*
Grade IV	1.237	0.956-1.601	0.106	1.334	0.991-1.797	0.058
**Clinical stage**						
Stage I	**Reference**			**Reference**		
Stage II	1.179	0.926-1.500	0.182	1.171	0.871-1.573	0.295
Stage III	1.661	1.310-2.105	<0.001*	1.832	1.372-2.446	<0.001*
Stage IV	3.056	2.291-4.077	<0.001*	3.519	2.511-4.933	<0.001*
**Tumor size**						
≤28	**Reference**			**Reference**		
29-69	2.071	1.879-2.282	<0.001*	2.681	2.366-3.039	<0.001*
≥70	3.051	2.726-3.415	<0.001*	4.110	3.577-4.722	<0.001*
**Metastasis**						
M0	**Reference**			**Reference**		
M1	0.998	0.732-1.360	0.989	1.058	0.759-1.476	0.739
**Radiotherapy**						
No/Unknown	**Reference**			**Reference**		
Yes	1.115	1.014-1.226	0.024	1.034	0.925-1.156	0.559
**Chemotherapy**						
No/Unknown	**Reference**			**Reference**		
Yes	0.745	0.688-0.807	<0.001*	0.772	0.704-0.845	<0.001*
**Historic stage**						
Localized	**Reference**			**Reference**		
Regional	1.539	1.212-1.954	<0.001*	1.842	1.371-2.474	<0.001*
Distant	2.265	1.522-3.371	<0.001*	2.652	1.688-4.166	<0.001*

HR, hazard Ratio; CI, confidence interval; *P < 0.05 were considered statistically significant.

### Construction of Prognostic Nomograms

**Figure 3:**
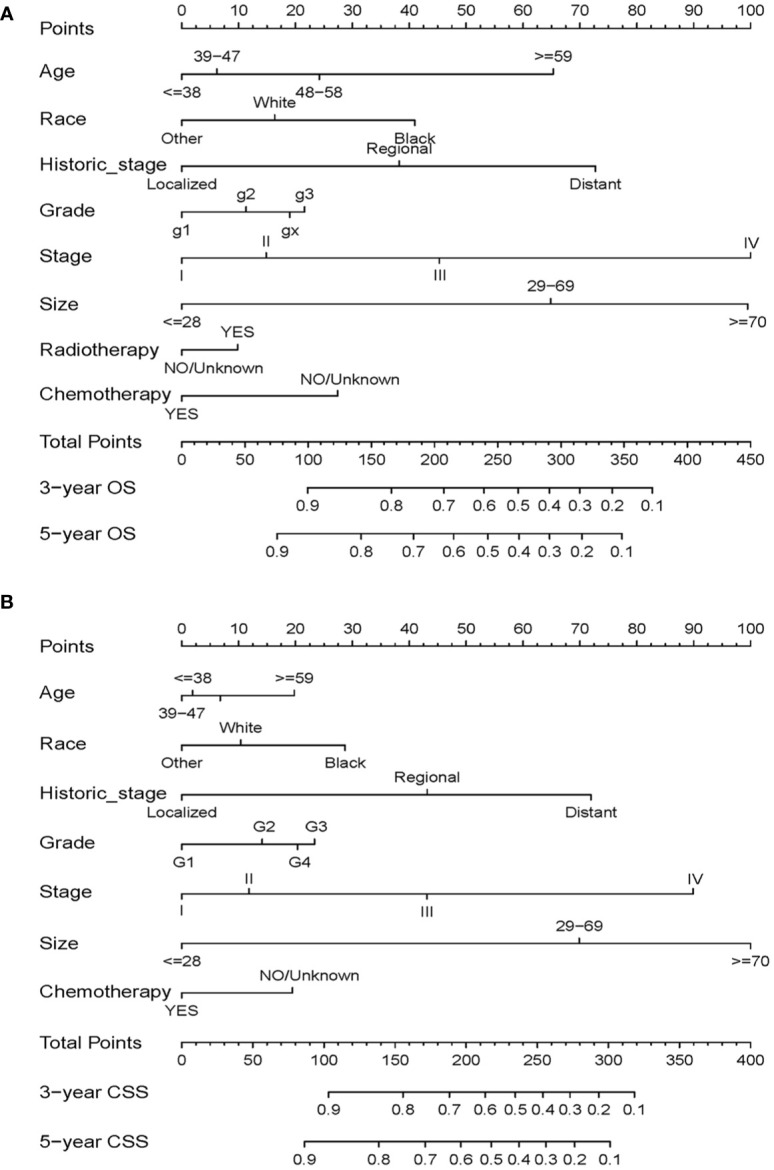
Nomograms for predicting 3- and 5-year OS **(A)** and CSS **(B)** in patients with cervical squamous cell carcinoma.

Based on the independent prognostic factors identified from the multivariate Cox regression analysis, nomograms were constructed to predict 3-year and 5-year survival in the training cohort for OS and CSS (**Figure 3**). Each variable is given a score on the “point axis” by its corresponding point. The scores of all variables are then added together to get the total score, then a vertical line is drawn down from the “total point axis” to the corresponding “survival axes” to estimate the predicted probability of 3- and 5-year survival. As shown in the nomogram for OS, clinical stage and tumor size made the largest contribution to the prognosis, followed by historical stage and age. The largest contribution to the prognosis in the CSS nomogram is tumor size, and followed by clinical stage

### Validation of the Nomograms

**Figure 4:**
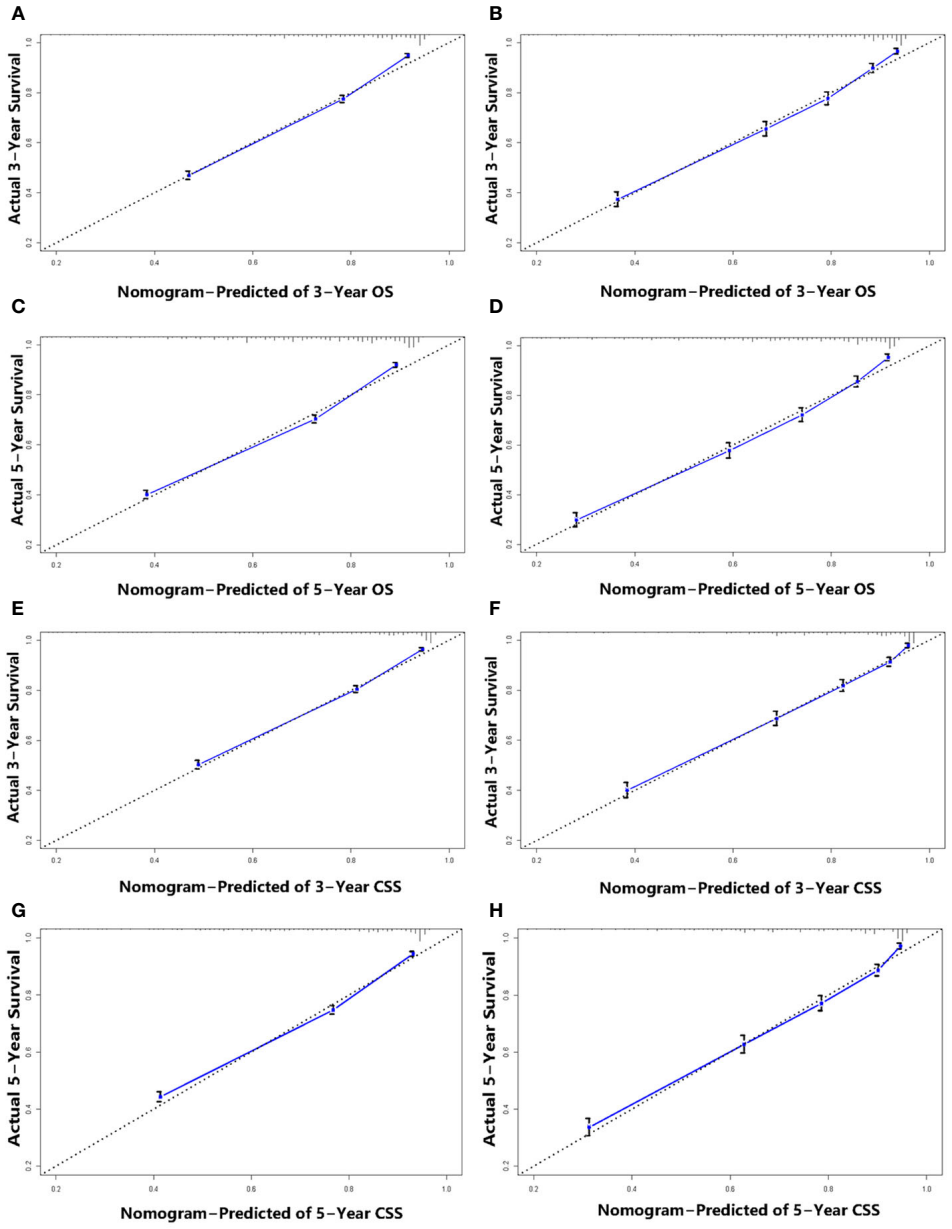
Calibration plots for 3-, and 5-year OS prediction for the training cohort **(A, C)** and validation cohort **(B, D)**. Calibration plots for 3-, and 5-year CSS prediction for the training cohort **(E, G)** and validation cohort **(F, H)**.

**Figure 5:**
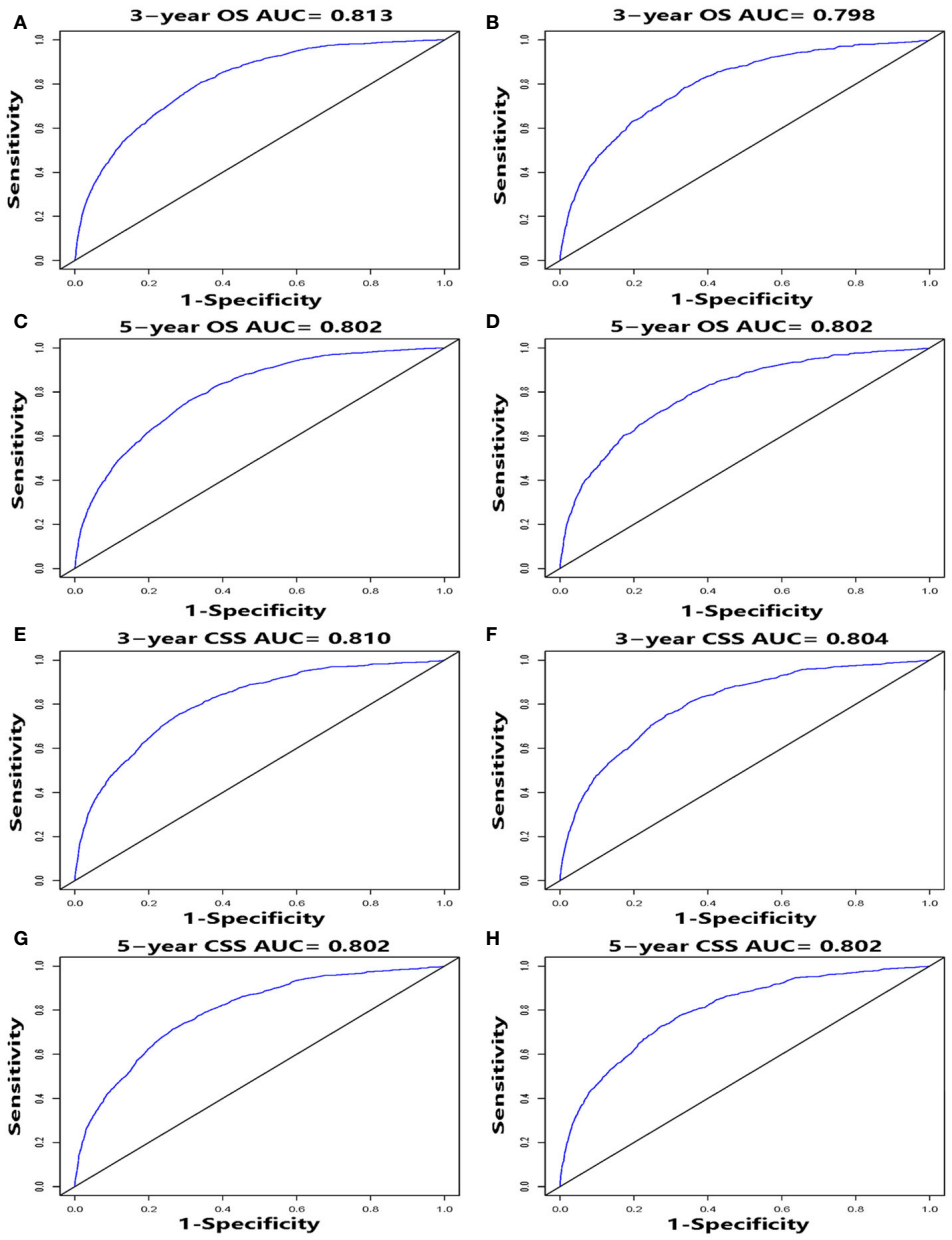
ROC curves, for 3- and 5-year OS and CSS, of the nomograms. ROC curves for 3- and 5-year OS in the training cohort **(A, C)** and validation cohort **(B, D)**. ROC curves for 3- and 5-year CSS in the training cohort **(E, G)** and validation cohort **(F, H)**.

We performed internal and external validation on the nomograms. For the internal validation of the nomogram, the prognosis for CSS gave a C-index of 0.786 (95% CI, 0.777–0.795) and for OS gave a C-index of 0.771 (95% CI, 0.762–0.780). For the external validation of the nomogram, prognosis for CSS gave a C-index of 0.797 (95% CI, 0.784–0.810) and for OS gave a C-index of 0.777 (95% CI, 0.765–0.789). The validation of these two nomograms demonstrated good predictive accuracy for both OS and CSS. The calibration plots for the nomograms showed that predictions of the 3-year and 5-year survival probability models of OS and CSS were almost consistent with actual observations, whether in the training cohort or in the validation cohort (**Figure 4**). ROC analysis showed that the AUCs for OS at 3 and 5 years were 0.813, 0.802 in the training cohort, and 0.798, 0.802 in the validation cohort, respectively. The AUCs for CSS at 3 and 5 years were 0.810, 0.802 in the training cohort, and 0.804, 0.802 in the validation cohort, respectively (**Figure 5**).

## Discussion

CSCC is one of the most common types of cancer in women and poses a serious threat to women’s health, causing about 273,200 deaths each year (20). While screening and HPV vaccination have remarkably decreased the incidence and mortality of CSCC in the United States, CSCC remains a significant public health challenge. Despite declining incidence and mortality rates, health disparities persist, as cancer screening is based on race, ethnicity, income and education (21). It is estimated that there will be approximately 13,800 newly diagnosed cases of CSCC in the United States in 2020, and 4,290 deaths are expected during the same period, almost the same number as in 2018 (20, 22). Clinical stage is the most important prognostic factor for CSCC. However, clinical stage does not fully reflect the biological heterogeneity of CSCC. From subclinical neoplasms to biologically aggressive carcinomas associated with metastatic spread and short patient survival, CSCC presents a highly variable course of disease (23). Patients with the same clinical stage may have markedly different treatment outcomes. Thus, it is necessary to determine effective prognostic indicators other than clinical stage. We attempted to construct and validate such clinical prognostic nomograms that assign predictions for OS and CSS of CSCC. The nomograms were derived from retrospectively collected data on 11,974 patients from the SEER dataset.

From the C-indexes of the nomogram based on internal (OS: 0.771, CSS: 0.786) and external (OS: 0.777, CSS: 0.797) cohorts obtained, the nomograms exhibited good predictive performance. Calibration curves, used to quantify how close predictions were to the actual outcome, showed that predictions were well calibrated (**Figure 4** **)**. Furthermore, the discriminatory capacity of the nomograms is also essential, and could be quantified by ROC curves. It was shown that the nomograms were well-discriminating models, based on AUC values (OS of the training cohort at 3 and 5 years: 0.813, 0.802; OS of the validation cohort at 3 and 5 years: 0.798, 0.802; CSS of the training cohort at 3 and 5 years: 0.810, 0.802; CSS of the validation cohort at 3 and 5 years: 0.804, 0.802) (**Figure 5**). The proposed nomograms provide more sophisticated tools for clinicians to help patients with CSCC obtain more personalized and tailored treatment to improve clinical prognosis.

Cox regression analysis showed that clinical stage and tumor size are independent prognostic factors for both OS and CSS, and these two factors are also the top two factors influencing the final risk score for OS and CSS in our nomograms, consistent with previous studies (5, 24). By using X-tile software, our results showed that 28 mm and 69 mm tumor size were the optimal cutoff points. Tumors between 29 – 69 mm and ≥ 70 mm had significantly lower survival rates than those ≤ 28 mm (p < 0.05, **Figure 2D**). Our conclusion is similar to that of other studies. Tumor size is an important prognostic factor for CSCC, especially in the early stages (25). In former staging systems, a tumor size of 4 cm was used as the cut-off to classify stage IB patients into IB1 (≤ 4 cm) and IB2 (> 4 cm). In the revised FIGO 2018 staging system, every 2 cm increase in tumor size is associated with an increase in sub-stage, and patients with stage IB tumors are further divided into three sub-stages: stage IB1 (< 2 cm), stage IB2 (2 – 3.9 cm) and stage IB3 (≥ 4 cm). There have been studies demonstrating significant differences in the survival rate between FIGO 2018 stage IB1 and IB2 disease (6, 26, 27), suggesting that the effect of tumor size can be further subdivided to improve survival discrimination for stage IB patients. As expected, clinical stage and tumor size contribute the most to the final risk score, but our goal was to look for other important prognostic factors to establish a more complete prognostic evaluation scheme.

CSCC is more common in middle-aged and older women. By Cox regression analysis, we identified age as an independent risk factor for OS and CSS. The X-tile program was then used to assess the optimal cut-off point(s) for age at diagnosis and found to be 38, 47, and 58 years (**Figure 2A**). Risk increased with age, and patients older than 58 years of age were more likely to have poor survival (**Figure 2B**). In the nomograms, the contribution of age to the final risk score was ranked fourth for OS (**Figure 3A**), but sixth for CSS (**Figure 3B**). In other words, the negative effect of age was more pronounced in OS than in CSS. This difference has been associated with degenerative changes in all aspects of organ function, an increased prevalence of multiple comorbidities and undertreatment in older patients (28–30). However, the effect of age on survival in patients with cervical cancer remains controversial. Some reports have been published that support our conclusion that age is an adverse prognostic factor for cervical cancer (30–34). In contrast, other studies suggest that younger patients have worse prognoses and lower survival rates (35), especially in the more advanced stages (36, 37). One likely reason is that younger women have a higher rate of cervical adenocarcinoma (38, 39), which has a poorer prognosis and is harder to detect than squamous cell carcinoma (40, 41). However, cervical adenocarcinoma was not included in our data analysis and discussion. In addition, other studies have reported that there is no significant difference in survival rates between older and younger women (42–44), but the premise of these studies was that all subjects, regardless of age, received aggressive treatment, and the reality was much more complicated. Older people are known to be less likely to receive aggressive treatment, and they tend to refuse it. Therefore, based on the above analysis, it is feasible and necessary to take age into account when analyzing the prognosis of CSCC.

Radiotherapy and chemotherapy are definitive treatments for CSCC. Our results show that radiotherapy and chemotherapy are independent predictors for OS, whereas radiotherapy can be excluded for CSS. Moreover, our nomogram reveals an interesting phenomenon: for OS, radiotherapy leads to poor prognosis. Therefore, it can be inferred that the side effects of radiotherapy may be detrimental to the long-term survival of CSCC patients. Radiotherapy is a common treatment for CSCC, and its most burdensome toxicities usually do not manifest until several years after treatment (45). Many studies have reported its deleterious effects on patients, including sexual dysfunction (46–48), urinary and intestinal dysfunction (49, 50), adverse psychological consequences (51–53), and increased risk of secondary malignancies (54–57). At the same time, studies have found that radiotherapy-based patients tend to have poorer prognosis with younger age (34, 58), which may be related to tumor-related leukocytosis (TRL) (58, 59), the level of sex hormones, such as estrogen (34, 60), and their receptor status (61). In addition, there is evidence that patients receiving concurrent chemoradiotherapy had better prognosis and higher OS compared with radiotherapy alone, both in early (62–64) and advanced (65–67) stages. At the same time, some studies have shown that preoperative or postoperative combination chemoradiotherapy, neoadjuvant chemotherapy or immunotherapy can improve patient progression-free and overall survival (62, 63, 66, 68–77). Despite these harmful effects, radiotherapy is still an effective treatment for CSCC. Therefore, when we decide on treatment for CSCC, we need to balance treatment outcomes, survival and reducing long-term adverse side effects in order to achieve the optimal therapeutic effect.

In our nomograms for predicting OS and CSS, the other predictors of reduced survival in CSCC patients involved race, advanced tumor grade, and higher historical stage. This result is consistent with many previous reports, suggesting that these three factors are independent predictors of survival in CSCC patients. In the United States, black women have a lower survival rate than white women (78, 79). Tumor grade and historical stage are also intrinsic characteristics of tumors and have been shown to be independent prognostic factors in CSCC patients (80–82). Recently, nomograms have been developed for the prediction of cervical cancer (11, 12, 14, 83). However, there are few nomograms specifically designed for patients with CSCC. The present study is the first to analyze the prognosis of CSCC patients and establish nomograms based on the SEER database. In this study, we selected a larger time span, so more patients could be included. At the same time, we selected and evaluated many influencing factors. While we did not include all the factors that might make sense because it would be too much work and impractical to do, we were able to ensure that all factors that ultimately comprised the nomogram were significant.

This study has the following limitations. First, this study did not involve cervical adenocarcinoma, although it has a high incidence and poor prognosis in young women (38–41). For cervical cancer patients, cervical squamous cell carcinoma accounts for about 90% of all cases, so we constructed a prognostic nomogram for patients with cervical squamous cell carcinoma. Second, due to the long-time interval of this study, there were inevitably some biases and missing data. As this was a retrospective study, there may be inherent flaws in retrospective data collection. We excluded patients with missing data when collecting variables, leading to a selection bias. Due to the fact that the database itself does not include some important tumor-related information, coupled with the limitations of censored data, practical operation and workload, we were unable to include more possible related factors, such as lymph vascular space involvement (LVSI), specific tumor markers, lymph node status, depth of tumor invasion, neoadjuvant chemoradiation therapy, and immunotherapy. According to known studies in literature, these factors were closely related to the prognosis of CSCC patients (6, 24, 63, 67, 68, 81, 84), but due to the limitations of the database and methods, they were not included in this study. Third, we randomly divided the patients into the training cohort and the validation cohort at a ratio of 2:1, constructed the nomogram, and performed internal and external validation, and the C-indexes and AUC values were relatively high. However, the data we used to build the model and calibrate the model came from the same database, imposed certain limitations on the suitable range of our model. Therefore, in subsequent work, we will use other databases in the United States and other countries in an attempt to improve the model. Finally, our nomograms have not been tested in real clinical trials, so their accuracy and practicability are still up for debate. Validation of our nomograms through randomized clinical trials will be the gold standard for testing their performance.

## Conclusion

In conclusion, we used the SEER database to analyze prognostic data for CSCC patients, identified independent prognostic factors, and constructed nomograms for estimating the 3- and 5-year OS and CSS. Internal and external validation showed that the model has satisfactory predictive performance and may be considered as a reliable tool to predict prognosis. However, its clinical utility has yet to be evaluated in other databases and randomized clinical trials.

## Data Availability Statement

The original contributions presented in the study are included in the article/supplementary material. Further inquiries can be directed to the corresponding author.

## Author Contributions

ZL and YL were major contributed in the study selection, data extraction, statistical analyses and manuscript writing. BC and QZ: provided useful suggestions in methodology. YC: designed and instructed the research. All authors contributed to the article and approved the submitted version.

## Funding

This study was funded by the Shantou Medical and Health Plan (grant No.180404094011013).

## Conflict of Interest

The authors declare that the research was conducted in the absence of any commercial or financial relationships that could be construed as a potential conflict of interest.
